# Study on Concrete Deterioration in Different NaCl-Na_2_SO_4_ Solutions and the Mechanism of Cl^−^ Diffusion

**DOI:** 10.3390/ma14175054

**Published:** 2021-09-03

**Authors:** Fei Zhang, Zhiping Hu, Feng Wei, Xin Wen, Xiaoguang Li, Li Dai, Long Liu

**Affiliations:** 1 Department of Civil Engineering, School of Civil Engineering, Chang’an University, Xi’an 710064, China; zhangfei@yulinu.edu.cn (F.Z.); 2017028001@chd.edu.cn (X.W.); gxleee@chd.edu.cn (X.L.); 2019128040@chd.edu.cn (L.D.); liulong9158@163.com (L.L.); 2 Department of Safety Engineering, School of Energy Engineering, Yulin University, Yulin 719000, China; 3 Department of Civil Engineering, School of Civil Engineering, Yulin University, Yulin 719000, China; wf_889@163.com

**Keywords:** mechanical property, diffusion rate, pore structure, interfacial transition zone, microstructure

## Abstract

The diffusion of sulfate (SO_4_^2−^) and chloride (Cl^−^) ions from rivers, salt lakes and saline soil into reinforced concrete is one of the main factors that contributes to the corrosion of steel reinforcing bars, thus reducing their mechanical properties. This work experimentally investigated the corrosion process involving various concentrations of NaCl-Na_2_SO_4_ leading to the coupled erosion of concrete. The appearance, weight, and mechanical properties of the concrete were measured throughout the erosion process, and the Cl^−^ and SO_4_^2−^ contents in concrete were determined using Cl^−^ rapid testing and spectrophotometry, respectively. Scanning electron microscopy, energy spectrometry, X-ray diffractometry, and mercury porosimetry were also employed to analyze microstructural changes and complex mineral combinations in these samples. The results showed that with higher Na_2_SO_4_ concentration and longer exposure time, the mass, compressive strength, and relative dynamic elastic modulus gradually increased and large pores gradually transitioned to medium and small pores. When the Na_2_SO_4_ mass fraction in the salt solution was ≥10 wt%, there was a downward trend in the mechanical properties after exposure for a certain period of time. The Cl^−^ diffusion rate was thus related to Na_2_SO_4_ concentration. When the Na_2_SO_4_ mass fraction in solution was ≤5 wt% and exposure time short, SO_4_^2−^ and cement hydration/corrosion products hindered Cl^−^ migration. In a concentrated Na_2_SO_4_ environment (≥10 wt%), the Cl^−^ diffusion rate was accelerated in the later stages of exposure. These experiments further revealed that the Cl^−^ migration rate was higher than that of SO_4_^2−^.

## 1. Introduction

Chloride ion (Cl^−^) invasion and corrosion is one of the main causes for the deterioration of reinforced concrete structures in the marine environment [1,2,3]. In the ocean, Cl^−^ migrates to the surface of the internal steel bars through concrete pores, interfacial transition zones (ITZ), or cracks and destroys the passive film of the bars, which causes corrosion and reduces the structure’s service life [4,5,6,7,8,9]. Some rivers, salt lakes and saline (alkaline) areas contain higher concentrations of Na_2_SO_4_ and NaCl [10]. For example, the Cl^−^ content in rivers is about 19.0 g/L and the SO_4_^2^^−^ content is about 3 g/L; the Cl^−^ content in salt lakes is about 19–23 g/L, and the SO_4_^2^^−^ content is about 22.2–36.4 g/L [11]. Reinforced concrete structures in these environments can be eroded by both Na_2_SO_4_ and NaCl, which do not act independently during the erosion process, but rather their influence one another (i.e., via mutual interactions [12,13] or synergy [14]). The combined invasion and corrosion of Na_2_SO_4_ and NaCl exacerbate the deterioration of reinforced concrete more significantly than the effects of only NaCl corrosion [15,16,17,18], thus highlighting the complexity of Cl^−^ diffusion in concrete.

To date, numerous scholars have carried out extensive NaCl exposure tests to study the diffusion and microstructural involvement of Cl^−^ in concrete. Hussein et al. [19] studied the influence of an NaCl environment on the mechanical properties of concrete, and their results showed that the mechanical properties of concrete decrease following long-term NaCl corrosion. Martin-Perez et al. [20] described the diffusion rate of Cl^−^ in concrete after different corrosion times in the marine environment. Luping [21], Yuan [22], and Wang et al. [23] each found that after Cl^−^ enters concrete, a portion of these ions will react with cement hydration products, including tricalcium sulfate hydrate, to form Friedel’s salt in a process called “chloride bindin” [24]. The remaining portion is adsorbed on the surface of cement hydration products in a process called “physical chloride adsorption,” or generally considered part of the “chloride binding effect” [25,26]. Meanwhile, the free Cl^−^ remaining in the pore solution continues to diffuse into the interior through pores or cracks, ultimately accumulates around the steel bars [27]. When the Cl^−^ attached to the surface of the steel bars exceeds a certain concentration, electrochemical corrosion of the steel bar may occur [28], resulting in decreased bonding performance between the bar and the concrete [29].

Several scholars have also investigated the corrosion products, microstructure, mechanical properties, and mechanisms of concrete corrosion after Na_2_SO_4_ attack [30,31]. Shannag [32] conducted various Na_2_SO_4_ corrosion tests and found that as the Na_2_SO_4_ concentration in solution increased, the relative dynamic elastic modulus (RDEM) and compressive strength of concrete, and damage failure time all decreased significantly. Najjar [33] found that the deterioration of concrete’s mechanical properties after sulfate corrosion was mainly due to reactions between SO_4_^2^^−^ and the aluminates (e.g., tricalcium aluminate, tetra-aluminate, and calcium monothioaluminate) of cement hydration products in concrete to produce ettringite (Aft) [34,35,36]. Additionally, sulfate corrosion tests revealed that the corrosion product Aft could be distributed throughout the ITZ and the internal concrete pores [37]. After Na_2_SO_4_ eroded concrete under a dry-wet cycle, the evolution of Aft was studied via thermal difference-thermogravimetry [38]. Bassuoni [39] used scanning electron microscopy (SEM) to analyze corrosion product crystal stress produced by high concentration Na_2_SO_4_ corrosion concrete and found that it is higher than the tensile strength of the matrix, this caused new microcracks to appear in the concrete, which decreased the concrete’s mechanical properties [40]. Zhao [41] studied the microstructure and mineral composition of cast-in-situ concrete in saline soil environment after sulfate erosion. Through SEM, X-ray diffraction (XRD), and thermogravimetric (TG) analysis, it was determined that the corrosion products (i.e., Aft and Gyp) of sulfate erosion filled the internal pores of the concrete, thus blocking the diffusion pathway and improving the erosion ability of concrete.

According to a literature survey, there are more than 1000 salt lakes scattered in Northwest China [42], and the salt lakes contain high concentrations of sulfate and chloride salts. The reinforced concrete in these salt lakes is severely damaged because of the long-term erosion of Na_2_SO_4_ and NaCl. It is well established that, the erosion of concrete by chloride and sulfate involves complex physical and chemical reactions [43,44]. However, saline soil contains many sulfates and chlorides, which increases the complexity of Cl^−^ diffusion mechanism in concrete in a SO_4_^2^^−^/ Cl^−^ mixed environment. To date, research regarding the influence of sulfate in concrete on Cl^−^ migration has been relatively limited, and there is insufficient experimental data to verify the relevant theoretical models. Therefore, it is necessary to fill the knowledge gaps related to the mechanical properties, Cl^−^ diffusion rates, pores, and microstructure of concrete in NaCl-Na_2_SO_4_ environment. To probe the mixed SO_4_^2^^−^/Cl^−^ exposure experienced by reinforced concrete in saline soil environment, the appearance changes, weight loss, mechanical properties (e.g., compressive strength and RDEM), and fineness of the concrete were examined after exposure to various concentrations of NaCl-Na_2_SO_4_ solutions (5 wt% NaCl and Na_2_SO_4_ at 0, 2.5, 5, 10 or 15 wt%) for different periods of time (30, 60, 90, 180, and 360 d). This study aimed to understand the evolution of concrete structures during salt invasion, and the resulting chemical and physical changes. The experiments used NaCl-Na_2_SO_4_ solutions to corrode the concrete, and analyzed the resulting changes in the appearance, weight loss, and mechanical properties of the concrete, which revealed the influence of different Na_2_SO_4_ concentrations on Cl^−^ migration. A capacitance mercury porosimeter was used to study the pore structure of the concrete after solutions exposure. Finally, SEM and XRD were used to analyze concrete microstructural changes, as well as corrosion products, and the evolution of ITZ with increasing Na_2_SO_4_ concentration.

## 2. Materials and Test Procedures

### 2.1. Materials

Portland cement of P.O. 42.5 (Jidong Heidelberg Jingyang Cement Co., Ltd., Xianyang, China) was selected to prepare concrete cube specimens. The chemical composition of the cement is shown in Table 1. Crushed stone from a 10 mm sieve was used as coarse aggregate and natural river sand in Xingping, Shaanxi used as fine aggregate (fineness modulus 3.24 and moisture content 8%). In experiments, distilled water was used to make mixed concrete and solutions. Na_2_SO_4_ reagent (analytically pure, 99.5%) was obtained from Tianjin Beichen Fangzheng Reagent Factory. The matching ratios of concrete in the test are shown in Table 2.

### 2.2. Sample and Solution Preparation

The corrosion conditions of different NaCl-Na_2_SO_4_ solutions were simulated by preparing five corrosion-resistant rectangular plastic boxes to store different concentrations of NaCl-Na_2_SO_4_ solutions, named CS0, CS0.5, CS1, CS2, and CS3 (Table 3). These solutions were checked monthly during timed erosions to ensure that samples were completely immersed in solution. At the same time, solution concentrations in these cuboid boxes were calibrated to ensure that SO_4_^2^^−^ and Cl^−^ concentrations were constant.

Before pouring the concrete, coarse aggregate, river sand, and cement were weighed and mixed for 3 min. Then, distilled water was added, and the mixture stirred for 3 min. Finally, the concrete was poured into 100 × 100 × 100 mm and 100 × 100 × 300 mm samples. After pouring, samples were let to stand for 24 h, demolded with a pressure air gun, and then moved to a standard curing room (temperature 20 ± 2 °C and humidity 95%). After curing for 28 d, samples were placed into different solutions for infusion for different periods. Before foaming, 4 surfaces of a sample were covered with epoxy resin, leaving two opposite surfaces clear to ensure that SO_4_^2^^−^ and Cl^−^ infusion was one-dimensional.

### 2.3. Experimental Procedures

Concrete samples were put in these various solutions for 30, 90, 180, 270, and 360 d. The sample should be placed in an oven at 50 °C for 48 h before testing.

#### 2.3.1. Weight Compressive Strength and RDEM

A sample weight was measured with an electronic scale with an accuracy of 0.01 g. An HYE-2000BS electro-hydraulic servo pressure testing machine (Hebei Sanyu Testing Machine Co., Ltd., Hebei, China) was used to test the compressive strength of concrete samples, with the loading speed controlled at 5 kN/s and average value of the compressive strength of 3 samples taken as the concrete compressive strength. A NELD-DTV dynamic elastic modulus tester (Beijing Naerde Instrument Equipment Co., Ltd., Beijing, China) was used to test the dynamic elastic modulus of concrete samples and weights collected with an electronic scale with an accuracy of 0.1 g. Three identical samples were produced for each test and the average taken as the concrete RDEM, with the test frequency set to 3000–9000 Hz.

#### 2.3.2. Cl^−^ and SO_4_^2^^−^ Concentration

The concentration of Cl^−^ and SO_4_^2^^−^ was determined from powder extracted at different depths from concrete samples obtained depths using a percussion drill. Three holes were drilled in the resin-free surface, the powders mixed from different positions, sifted with a 0.63 mm sieve, and placed in an oven at 105 °C for 2 h until there is no significant change in the quality of the powders. A 2 g mass of each sample powder was mixed with distilled water and then left to dissolve its Cl^−^ and SO_4_^2^^−^ for 48 h. The resulting solutions were passed through filter paper to remove remaining solids. A CLU-W (Beijing Zhongke Road Construction Equipment Co., Ltd., Beijing, China) type Cl^−^ content rapid tester and Ultraviolet-visible spectrophotometer (Shimadzu Enterprise Management (China) Co., Ltd., Shanghai, China) were used to measure the concentration of Cl^−^ and SO_4_^2^^−^ in each sample. It should be noted that, due to the uneven distribution of aggregates in the concrete, at least three samples were measured to obtain relatively accurate Cl^−^ and SO_4_^2^^−^ results.

#### 2.3.3. SEM, EDS, and XRD Analyses

SEM/EDS analyses of concrete samples were obtained using a Sigma 300 field emission scanning electron microscope (Carl Zeiss AG, Oberkochen, Germany) and the samples were photographed and elementally analyzed at different magnifications. X-ray diffractometry was performed using a Bruker D8 Advance instrument (Bruker Corp., Billerica, MA, USA), with the voltage 40 kV, scanning speed 5°/min, and minimum current 100 mA. SEM/EDS test samples were produced by breaking a concrete sample, selecting a representative block, soaking it in absolute ethanol, and then placing it in an oven at 50 °C for 24 h. Powder samples for X-ray diffraction analysis were drilled 10 mm into a concrete sample surface with an electric hammer and filtered through a 0.075 mm sieve.

#### 2.3.4. Pore Analysis

**Figure 1:**
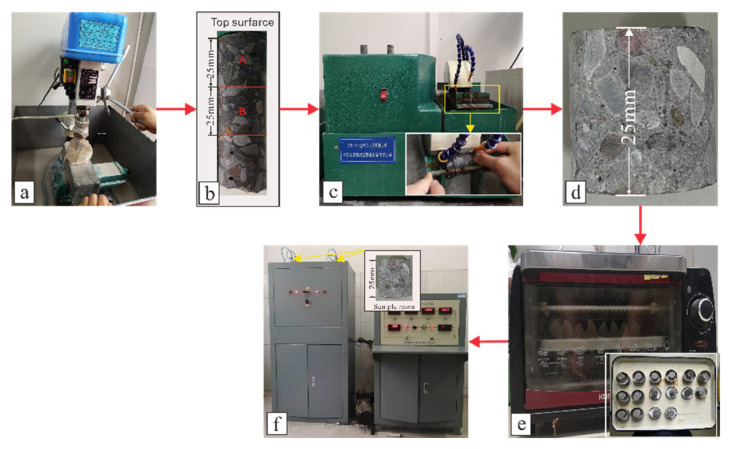
Porosity determination process: corebit, columnar, slicing, sample, mercury injection test (**a**–**f**, respectively).

Concrete pore analysis was performed using a YG-97A capacitive mercury porosimeter (Wuxi Huiao Instrument and Equipment Manufacturing Co., Ltd., Jiangsu, China) to measure the sample pore structures after 30, 180, and 360 d of corrosion and to 25 mm (Figure 1b, Position A) and 25–50 mm depth after 360 d (Figure 1b, Position B). First, a TZ-1 desktop rock core machine was used to drill a 25 × 75 mm long cylinder from a sample, a QP-100 desktop high-power slicer used to cut samples at 25 and 50 mm from the surface, and finally the sample placed in an oven at 50 °C for 8 h. After the sample had cooled, a YG-97A capacitive mercury porosimeter was employed to examine sample pores. The related process is shown in Figure 1.

## 3. Results

### 3.1. Weight Changes and Appearance of Performance

**Figure 2:**
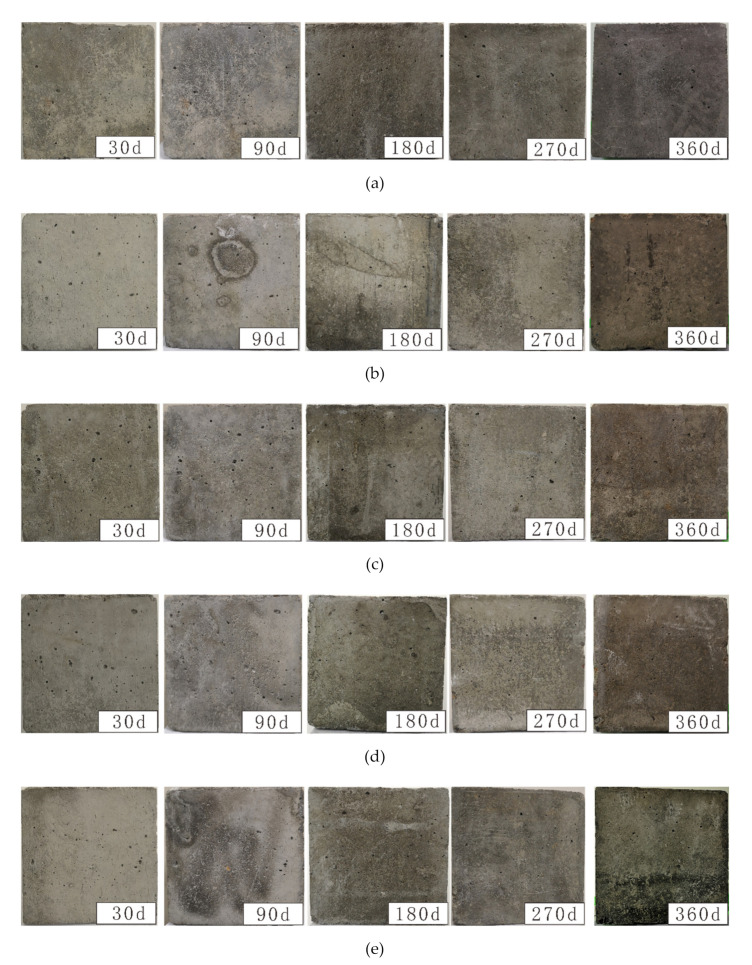
Changes of specimen appearance immersed in CSO, CS0.5, CS1, CS2, and CS3 (**a**–**e**, respectively).

**Figure 3:**
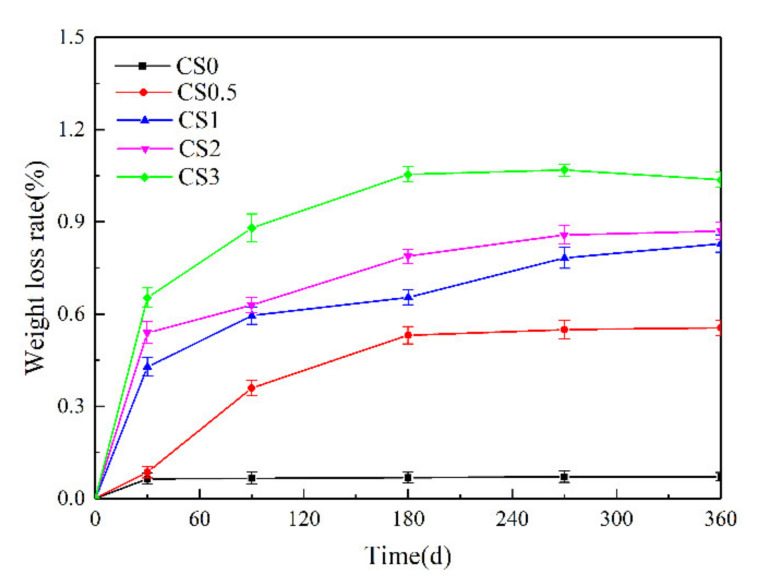
The changes of specimen weight versus time in different solutions.

After specimens were attacked for 30, 90, 180, 270, and 360 d, the sample weights were recorded. Photos of these samples after being attacked to different solutions for the designated periods of time are shown in Figure 2, and they reveal clear changes in sample appearance depending on their environment. The weight change rate was calculated according to the relationship: (W_t_ − W_0_) × 100%/W_0_ (where W_0_ is the weight before treatment, and W_t_ is the weight after exposure for a given periods t). The weight changes for different solutions are presented in Figure 3.

The observed changes in the concrete samples’ appearance after different corrosion times in solutions of 5 wt% NaCl and 0, 2.5, 5, 10, or 15 wt% Na_2_SO_4_ indicated that the surface pores in the CS0.5, CS1, CS2, and CS3 samples gradually increased with corrosion time. Simultaneously, the sharp shapes of cement paste around pores gradually adopted arc-shaped, and pore outer diameters gradually increased (Figure 2). In particular, as the Na_2_SO_4_ concentration in the mixed solution increased, the outer diameter of the pores changed significantly. Samples from the CS2 and CS3 solutions exhibited slight powder peeling after 270 d of corrosion, and a few small cracks appeared in the CS3 samples after 360 d. During corrosion, the surface pores on the CS0 samples did not change. Therefore, as the concentration of Na_2_SO_4_ increased on concrete surfaces during corrosion, the degradation rate gradually accelerated.

The weight of concrete is generally affected by corrosion products, cement hydration, and the flaking of concrete powder or fragments. The weight change of concrete samples after different lengths of time in various corrosion solutions indicated that as the Na_2_SO_4_ concentration increased, the specimen weights changed significantly (Figure 3). The weight of the CS0 samples increased slowly up to 30 d of corrosion and increased only slightly thereafter. The weights of the CS0.5, CS1, CS2, and CS3 samples, although the rate of increase became slower with longer corrosions. However, the CS2 and CS3 sample weights began to decline after 270 and 180 d exposure, respectively, and the CS3 sample weights decreased significantly. The weight change and appearance of these concrete samples showed similar results.

The changes in concrete specimens’ weights and appearances were mainly related to solution corrosion time and concentration gradient. Specifically, as the Na_2_SO_4_ concentration increased, SO_4_^2^^−^ migration into the concrete also increased. At this time, SO_4_^2^^−^ and Cl^−^ and other chemical reaction products migrated into the concrete, causing precipitates to accumulate, thereby affecting the concrete pores, ITZ, and microcracks. Overall, the weight and appearance of the corroded concrete over long periods of time changes significant.

### 3.2. Compressive Strength and RDEM

#### 3.2.1. Compressive Strength Changes and Failure Mode

**Figure 4:**
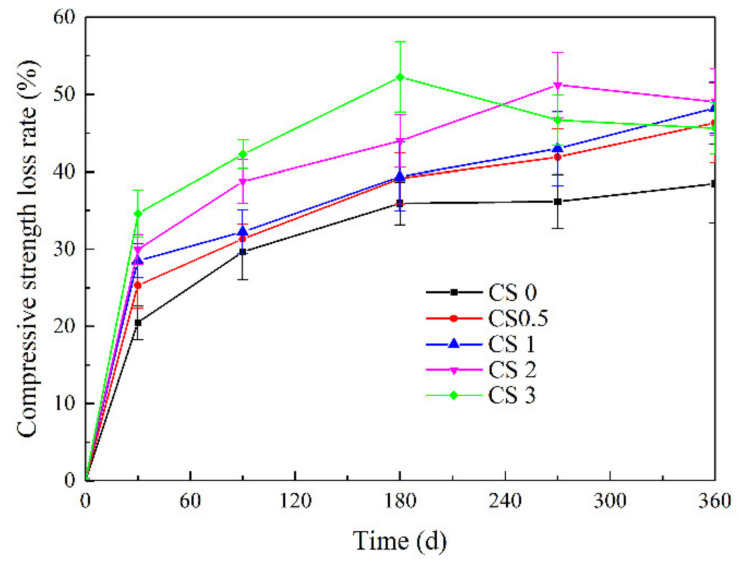
The loss rate of compressive strength of concrete specimens against corrosion time.

Uniaxial compressive strength is an important indicator for measuring the strength and damage characteristics of concrete materials in a corrosive environment and it can more intuitively reflect the relationship between the damage and the concentration and time of exposure to a corrosive solution. According to Equation (1), the loss of concrete compressive strength in different salt solutions was calculated.

(1) $$\Delta f_{c}\left(n\right)=\frac{f_{c,0}-f_{c,n}}{f_{c,0}}\times 100\%$$

where: ∆f_c_ (n) is the change in compressive strength of concrete after corrosion n d, %; ∆f_c,0_ is the value of 28 d compressive strength of concrete before corrosion, MPa; ∆f_c,n_ is after n days of corrosion compressive value of concrete, MPa;

The change in the compressive strength of concrete samples after corrosion in solutions of 5 wt% NaCl and 0, 2.5, 5, 10, and 15 wt% Na_2_SO_4_ for designated times were generally consistent regardless of the solutions composition (Figure 4). There were two stages, which involved rapid and slow increase, respectively. The CS2 and CS3 samples were attacked for 270 and 180 d, respectively, and then exhibited slow declining stage. The strength loss of the CS3 samples was greater than that in CS2, but for the CS0, CS0.5, and CS1 samples, there was no clear downward trend in terms of compressive strength by the end of corrosion periods. Similar to the previous results involving low Na_2_SO_4_ content (≤ 5 wt%), no significant changes were observed on sample surfaces (Figure 2). Therefore, when the Na_2_SO_4_ was high, the concrete suffered more serious damage, resulting in a downward trend in compressive strength.

**Figure 5:**
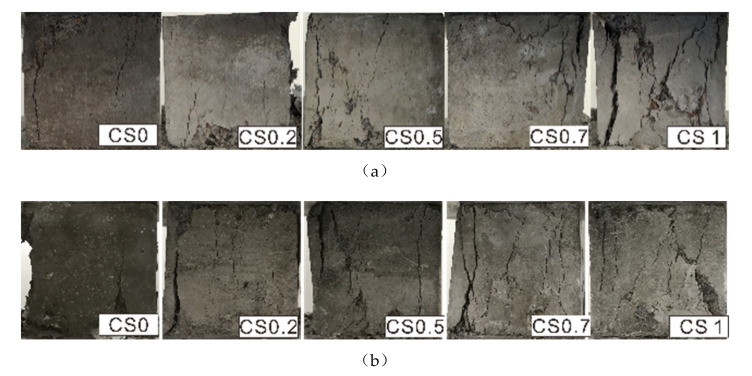
Appearance of compressive strength damage: eroded 180 d (**a**) and 360 d (**b**).

The observable damage changes after the compressive strength tests of samples after 180 and 360 d of salt corrosion showed that after 180, there were fine cracks at the CS0 samples edges (Figure 5). As the Na_2_SO_4_ concentration increased, surface cracks gradually emerged and became dense; these cracks further expanded and becoming thicker and longer. The position of these cracks migrated toward the center of the specimens, especially in CS3 solution, where the cracks developed an “X” shape, and a small amount of peeling appeared on the surfaces. After 180 or 360 d of exposure, changes in sample surface cracks in the CS0 solution were not very significant. As the Na_2_SO_4_ concentration increased, the surface cracks on the sample in the CS1 solution adopted a “V” shape, which shifted toward the center of the sample. There were many small cracks in the CS2 samples, which were cross-connected with each other. Numerous intersecting cracks appeared in the CS3 samples, and the surface peeling phenomenon relatively serious.

It was clear from these analytical results that changes the compressive strength mainly depended on the cement hydration, SO_4_^2^^−^ concentration in the salt solution, and attack time. Long-term attack led to changes in the concrete structure to a certain depth. As SO_4_^2^^−^ and Cl^−^ reacted with hydration products in concrete, they changed the distribution of pores, ITZ, and cracks inside the concrete, and this affected the compressive strength and damage morphology of the concrete.

#### 3.2.2. Relative Dynamic Modulus of Elasticity

Changes in the RDEM can somewhat reflect the integrity, compactness, and degree of damage to the. The RDEM changes in the concrete samples exposed to different solutions were calculated according to Equation (2).

(2) $$\Delta E_{n}=\frac{E_{n}}{E_{0}}$$

where: ∆E_n_ is the change value of concrete dynamic elastic modulus after corrosion n d; E_0_ is the dynamic elastic modulus of concrete after 28 d of standard curing before corrosion, MPa; E_n_ is the dynamic elastic modulus of concrete after corrosion for n days, MPa.

**Figure 6:**
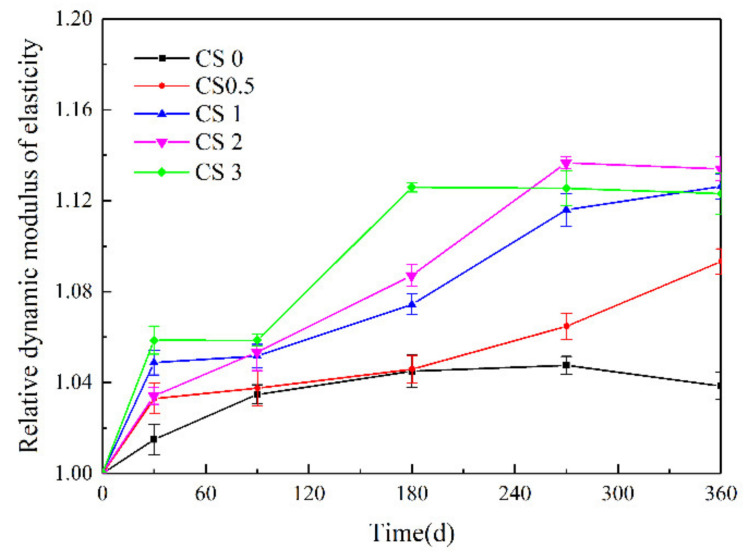
Relative dynamic modulus of elasticity of concrete specimens against corrosion time.

As the corrosion time increased, its RDEM increased but the rate of increase tended to decelerate (Figure 6). The RDEM of samples in the CS0 solution was generally lower than that of samples exposed to Na_2_SO_4_. As the Na_2_SO_4_ concentration increased, RDEM decreased and the CS3 samples exhibited a downward trend. This indicted that the increased RDEM of the concrete during the initial salt corrosion stage was mainly due to cement hydration and the filling of pore structures upon NaCl and Na_2_SO_4_ corrosion, which increased concrete’s integrity and density. In concentrated Na_2_SO_4_ solution, as the concrete structure (to a certain depth) was damaged by corrosion products, new pores or cracks were generated in the samples, which reduced their RDEM. These results showed that the RDEMs of concrete in different salt solutions were basically consistent with the changes in compressive strength, however, the impact of Na_2_SO_4_ on compressive strength was more significant than on RDEM.

### 3.3. Chlorine Salt and Sulfate Concentration

**Figure 7:**
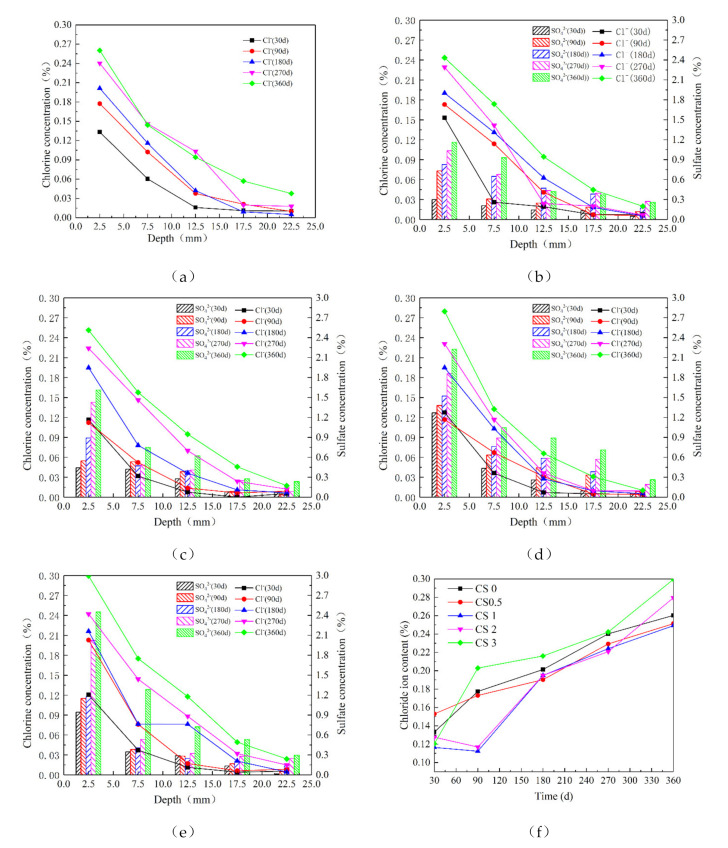
Results of Cl^−^ and SO_4_^2^^−^ concentrations: CS0, CS0.5, CS1, CS2, and CS3 (**a**–**e**, respectively), and (**f**) ion content at 2.5 mm.

The concentrations of NaCl and Na_2_SO_4_ in samples at various depths during corrosion with different solutions were examined. The results involving Cl^−^ and SO_4_^2^^−^ ions in the same solution were gathered to make a clear comparison (Figure 7).

For concrete in 5 wt% NaCl and 0, 2.5, 5, 10, and 15 wt% Na_2_SO_4_ solutions, it was clear that the Cl^−^ and SO_4_^2^^−^ in these solutions were relatively more concentrated near the sample surfaces, and their concentrations decreased rapidly with increasing depth from the sample surface. In addition, as the concentration of Na_2_SO_4_ increased, SO_4_^2^^−^ penetration into each sample became deeper, and the resulting concentration higher. The influence of Na_2_SO_4_ concentrations on the migration of Cl^−^ was further explored in terms of Cl^−^ changes at 2.5 mm depth in samples exposed to different solutions and corrosion times (Figure 7f). When the Na_2_SO_4_ concentration was ≤10 wt%, the Cl^−^ content of the solution containing Na_2_SO_4_ was lower than that of the CS0 solution as a whole. After 300 d of salt solution exposure, the CS2 sample’s Cl^−^ content was higher than that of the CS0 samples. The Cl^−^ content in the CS3 solution was lower than in the CS0 sample before 30 d of exposure, at which point the Cl^−^ content exceeded that of the CS0 samples. This result showed that low-concentration Na_2_SO_4_ solution inhibited Cl^−^ migration; as the Na_2_SO_4_ concentration and exposure increased, the higher concentration of Na_2_SO_4_ promoted Cl^−^ migration.

### 3.4. Microstructural and Mineral Analytical Results

The corrosion products and microstructures produced by different solutions after 30, 90, 180, 270, and 360 d of attack were examined by microstructural and mineral analyses of the corresponding concrete samples.

#### 3.4.1. XRD Test

**Figure 8:**
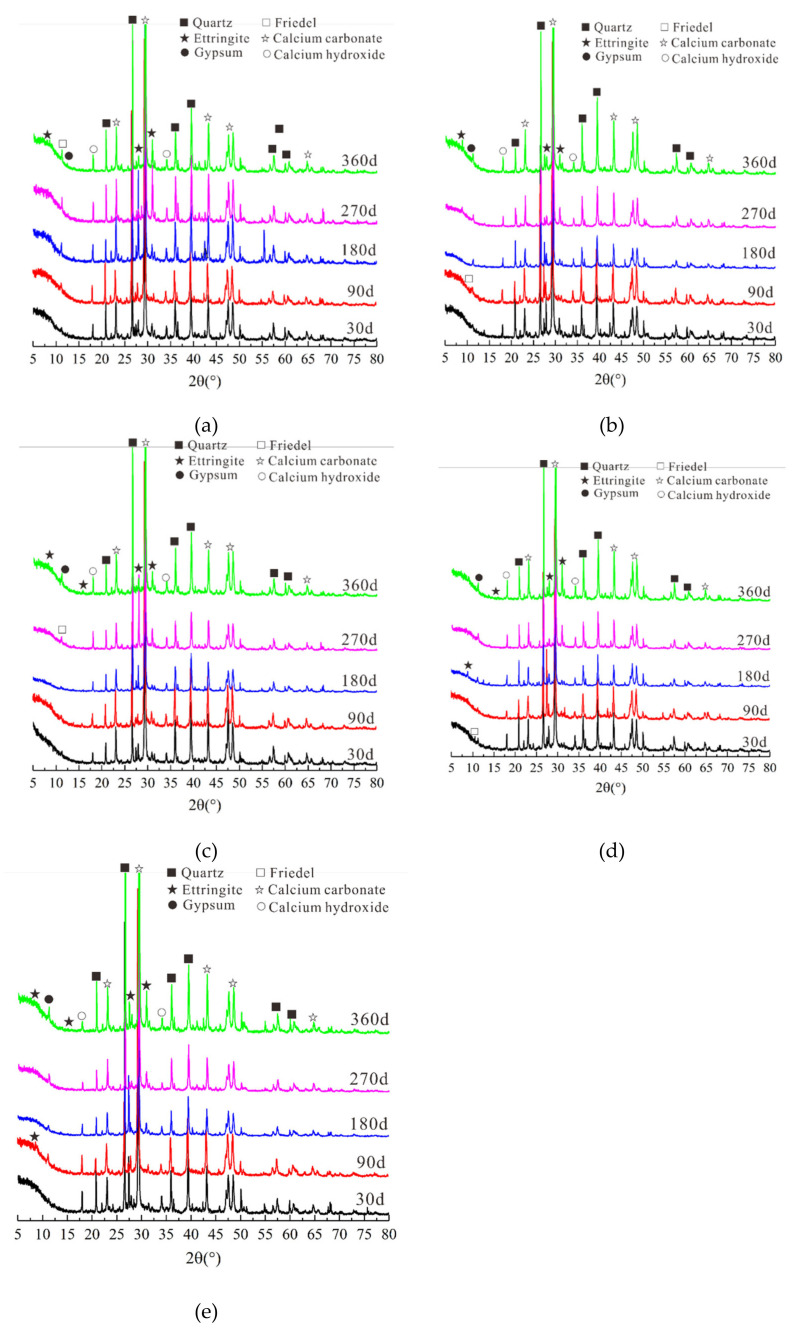
Mineral analysis results after corrosion for the specimen: (**a**) CS0, (**b**) CS0.5, (**c**) CS1, (**d**) CS2, (**e**) CS3.

The X-ray diffraction patterns of concrete sample attacked to NaCl-Na_2_SO_4_ solutions for various times indicated that the corrosion products were generally the same regardless of the salt concentrations and exposure times, mainly because of the differences in peak diffusion and reaction intensity (Figure 8). As the attack time increased, the intensity of the Friedel’s salt diffraction peak in the CS0 sample gradually increases, but the calcium hydroxide (CH) diffraction peak gradually decreased because the reaction between Cl^−^ ions and cement hydration products (single-sulfur hydrated calcium sulphoaluminate (AFm)) generate Friedel’s salt and release SO_4_^2^^−^ at the same time [45]. With higher Na_2_SO_4_ concentration and longer times, AFm and Friedel’s salt reacted with SO_4_^2−^ to generate stable corrosion products Aft [46]. Additionally, Gyp crystals were formed after long-term erosion in Na_2_SO_4_ solution. Therefore, the diffraction peaks of Aft and Gyp in these samples gradually increased, whereas the intensity of Friedel’s salt and CH diffraction peaks gradually decreased. The Friedel’s salt diffraction peak was present in the diffraction pattern of the late-stage CS0.5 sample and early-stage CS1 and CS2 samples, but it did not appear in CS3 samples with high Na_2_SO_4_ concentration. This was mainly because the migration rate of Cl^−^ in concrete was >100 times that of SO_4_^2^^−^. After SO_4_^2^^−^ diffuses into the concrete, more Friedel’s salt was formed, and the small number of times that of SO_4_^2^^−^. After SO_4_^2−^ diffused into the concrete, more Friedel’s salt was formed, and the small amount of SO_4_^2−^ only reacts with a portion the available Friedel’s salt to form Aft after it migrates into the concrete. In the CS2 samples, the Aft diffraction peak appeared relatively early and peak intensity relatively was relatively strong. Aft also appeared early in the CS3 samples. With extended exposure time, the CH and Aft peaks gradually disappeared, and the Gyp peak increased, which indicated that during concrete exposure to Na_2_SO_4_, Cl^−^ and SO_4_^2^^−^ first react with Afm to form Friedel’s salt and Aft, and then Friedel’s salt reacts with SO_4_^2^^−^ to form Aft. Therefore, the peak intensities of Friedel’s salt and CH gradually decreases with the increase NaCl-Na_2_SO_4_ concentration and with the extension of erosion time. At this time, the peak corresponding to Aft gradually increased. However, if the concentration was too high or the attack too long, the peak associated with Friedel’s salt in the concrete disappeared, and that of Gyp gradually increased.

#### 3.4.2. SEM and EDS Analyses

**Figure 9:**
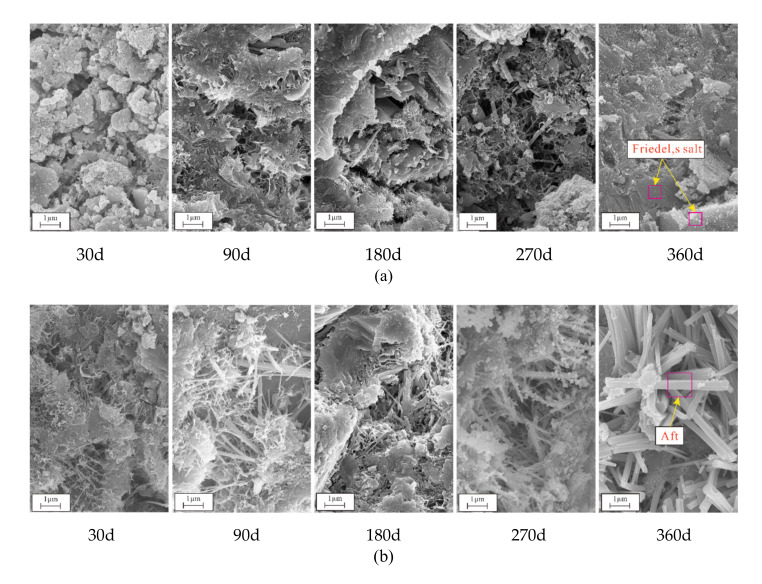

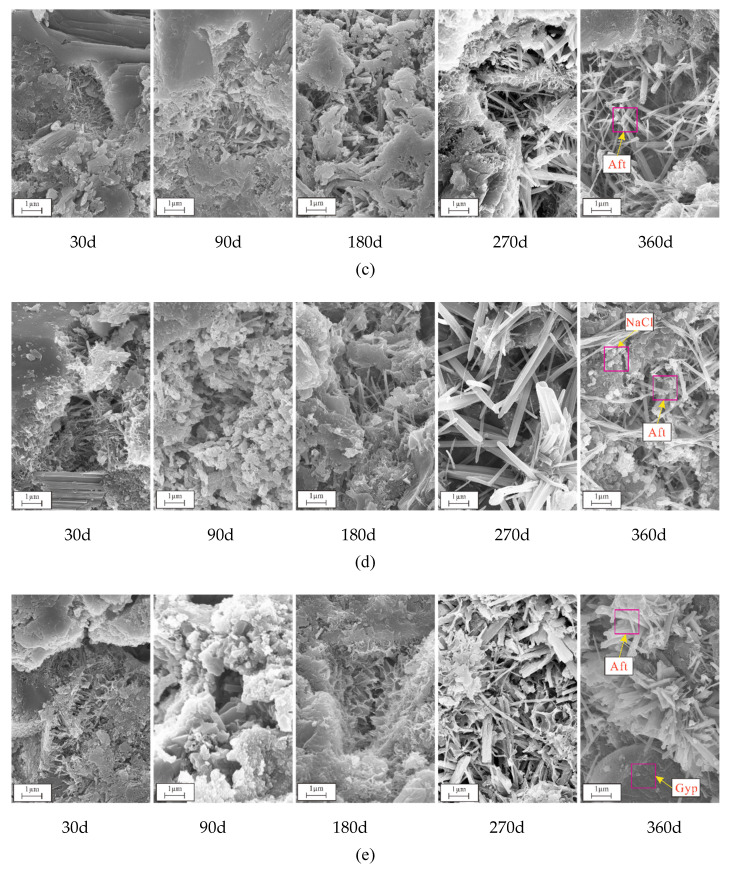
SEM images of samples in different erosion solutions: (**a**) CS0, (**b**) CS0.5, (**c**) CS1, (**d**) CS2, (**e**) CS3.

**Figure 10:**
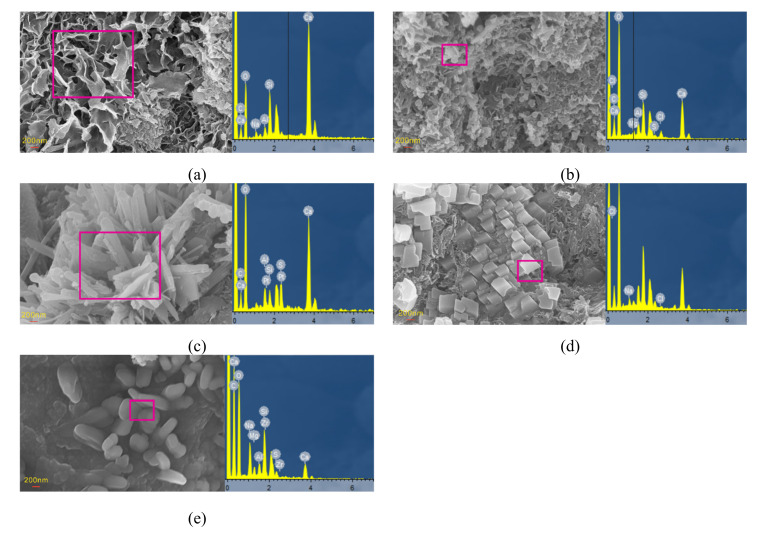
Microstructures and EDS results of selected region of concrete after corrosion for the specimen: (**a**) C-S-H, (**b**) Friedel’s salt, (**c**) Aft, (**d**) NaCl, (**e**) Na_2_SO_4_.

**Figure 11:**
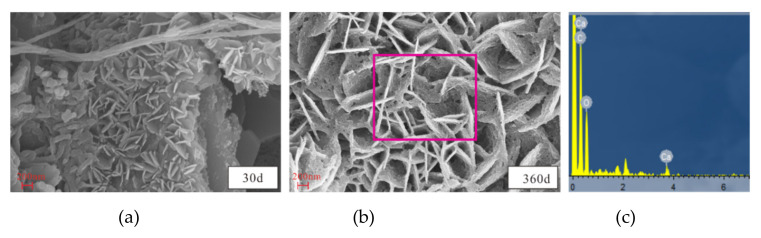
Picture of Ca (OH)_2_ microstructure and EDS in CS3 solution: (**a**) 30d, (**b**) 360d, (**c**) EDS.

**Figure 12:**
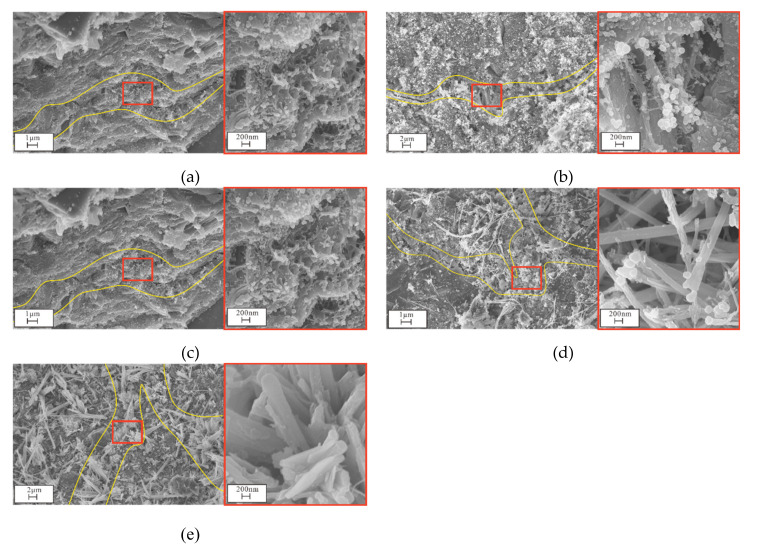
Crack evolution of concrete after being eroded by solutions of different concentrations for 360 days: (**a**) CS0, (**b**) CS0.5, (**c**) CS1, (**d**) CS2, (**e**) CS3.

**Figure 13:**
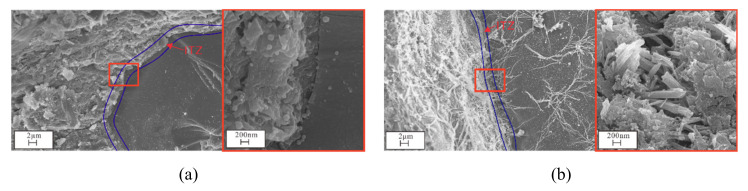
ITZ evolution in CS0 and CS3 solution after 360 days of erosion: (**a**) CS0, (**b**) CS3.

Examination of SEM and EDS results for samples after different exposure times enabled analysis of the microstructure and composition diagrams after salt solution exposure for various periods (Figure 9, Figure 10, Figure 11, Figure 12 and Figure 13). The results showed that the main corrosion products were Friedel’s salt (3CaO∙Al_2_O_3_∙CaCl_2_∙10H_2_O), Aft (3CaO∙Al_2_O_3_∙3CaSO_4_∙32H_2_O) and Gyp (CaSO_4_∙2H_2_O), and main corrosion reactions are as follows in Equations (3)–(9) [47,48,49,50,51,52].

(3) $$\mathrm{CaO}\cdot {\mathrm{Al}}_{2}O_{3}\cdot 3{\mathrm{CaSO}}_{4}\cdot 12H_{2}O+2{\mathrm{Cl}}^{-}\to 3\mathrm{CaO}\cdot {\mathrm{Al}}_{2}O_{3}\cdot {\mathrm{CaCl}}_{2}\cdot 10H_{2}O+{\mathrm{SO}}_{4}{}^{2-}+\;2H_{2}O$$

(4) $$3\left(2\mathrm{CaO}\cdot {\mathrm{SiO}}_{2}\right)+4H_{2}O\to 3\mathrm{CaO}\cdot {\mathrm{Al}}_{2}O_{3}\cdot 6H_{2}O+\mathrm{Ca}{\left(\mathrm{OH}\right)}_{2}$$

(5) $$3\mathrm{CaO}\cdot {\mathrm{Al}}_{2}O_{3}+6H_{2}O\to 3\mathrm{CaO}\cdot {\mathrm{Al}}_{2}O_{3}\cdot 6H_{2}O$$

(6) $${\mathrm{SO}}_{4}{}^{2-}+\mathrm{Ca}{\left(\mathrm{OH}\right)}_{2}+2H_{2}O\to {\mathrm{Ca}}_{2}{\mathrm{SO}}_{4}\cdot 2H_{2}O+2{\mathrm{OH}}^{-}$$

(7) $$3\left({\mathrm{CaSO}}_{4}\cdot 2H_{2}O\right)+3\left(3\mathrm{CaO}\cdot {\mathrm{Al}}_{2}O_{3}\cdot 6H_{2}O\right)+26H_{2}O\;\to 3\mathrm{CaO}\cdot {\mathrm{Al}}_{2}O_{3}\cdot 3{\mathrm{CaSO}}_{4}\cdot 32H_{2}O$$

(8) $$3\left({\mathrm{CaSO}}_{4}\cdot 2H_{2}O\right)+{\mathrm{SO}}_{4}{}^{2-}+{\mathrm{Ca}}^{+}+22H_{2}O\;\to 3\mathrm{CaO}\cdot {\mathrm{Al}}_{2}O_{3}\cdot 3{\mathrm{Ca}}_{2}\mathrm{SO}4\cdot 32H_{2}O+2{\mathrm{Cl}}^{-}$$

(9) $$3\mathrm{CaO}\cdot {\mathrm{Al}}_{2}O_{3}\cdot {\mathrm{CaCl}}_{2}\cdot 10H_{2}O+3\mathrm{CaO}\cdot {\mathrm{Al}}_{2}O_{3}\cdot 6H_{2}O+2\mathrm{Ca}{\left(\mathrm{OH}\right)}_{2}+24H_{2}O\to \;3\mathrm{CaO}\cdot {\mathrm{Al}}_{2}O_{3}\cdot 3{\mathrm{CaSO}}_{4}\cdot 32H_{2}O$$

Figure 9 presents the microstructures of samples after attack to various NaCl-Na_2_SO_4_ solutions for various. The CS0 samples had a loose granular structure when exposed for 30 d, and a small amount of rod-like and net-like structures gradually appeared over time (Figure 9). This net-like structure was analyzed by EDS and determined to be calcium-silicate-hydrate (C-S-H, Figure 10a). There were more irregular flake structures in the sample after 360 d of attack, which were ascribed as Friedel’s salt (Figure 10b). As the Na_2_SO_4_ concentration and exposure time increased, the number of rod-like structures gradually increased, became denser, and filled the pores and cracks. EDS analysis showed that the main elements were C, Si, Ca, Al, and S, with the latter three attributed to the Aft crystals (Figure 10c). It was further discovered that there were massive, flaky crystals in samples attacked to different solutions, which were determined to be NaCl (Figure 10d) and CH crystals (Figure 11). After 360 d of attack, there were petal-like crystals in CS2 and CS3 samples, which were assigned to be Na_2_SO_4_ crystals based on the contained elements (Figure 10e). The microstructure of the CH crystals obtained following exposure to the CS3 solution exhibited relatively complete sheet-like structure after 30 d, and they were distributed across the sample; after 360 d, more pores appeared on the CH crystal surfaces, and the surrounding area were damaged.

Sample crack evolution following attack to different salt solutions for 360 d revealed that the microstructure of the CS0 samples was relatively finely distributed, and there were numerous C-S-H network gel structures in the cracks (Figure 12). As the Na_2_SO_4_ concentration increased, sample cracks gradually widened, and C-S-H was gradually replaced by Aft. Cracks in the CS2 samples were filled by Aft and new cracks generated. Cracks in CS3 samples were completely filled by Aft, and new cracks were generated. Cracks in the CS3 sample were completely filled by Aft, which adopted a popcorn shape. Meanwhile, new microcracks that were connected to one another appeared in the CS2 and CS3 samples. It is well known that the ITZ is the weakest part of the concrete and that it is also favorable for ion diffusion. When there is a small crystal stress, the ITZ can be damaged, leading to extension and widening of ITZ cracks. The ITZ microstructure after 360 d of exposure in the CS0 and CS3 samples showed that (i) the mortar and aggregate were tightly connected, (ii) there were more rod-shaped Aft crystals in the ITZ in the CS3 samples, and (iii) the ITZ was wider than that of the CS0 sample (Figure 13).

These results showed that when Na_2_SO_4_ was present in the salt solution, the corrosion products altered the concrete microstructure. With low Na_2_SO_4_ concentration or short exposure, the internal pores and cracks of the concrete were filled with corrosion products, which improved the mechanical properties of the concrete and increased its density. With higher Na_2_SO_4_ concentration or longer attack, the corrosion products Aft and Gyp gradually accumulated in pores, cracks, and the ITZ. Additionally, C-S-H changed from a block to a honeycomb structure, with had lower strength. At the same time, the corrosion products generated greater crystal stress, resulting in damage to the internal pores, cracks, and ITZ of the concrete. This also produced new microcracks that crossed one another, thus promoting Cl^−^ and SO_4_^2^^−^ diffusion. Simultaneously, CH crystals were eroded and destroyed by the Na_2_SO_4_, resulting in accelerated damage to the concrete’s mechanical properties.

### 3.5. Pore Analysis

The interior of concrete is a multiphase-porous composite material comprising of pores of various sizes and types. The pore structure is directly related to ion migration and the material’s mechanical properties. A YG-97A capacitive mercury porosimeter was used to analyze the concrete pore structure of samples that had been attacked to NaCl-Na_2_SO_4_ solutions for 30, 180, and 360 d. For this purpose, concrete pores were categorized into three types: small pores (<0.05 µm), middle pores (0.05–10 µm), and large pores (10–63 µm).

**Figure 14:**
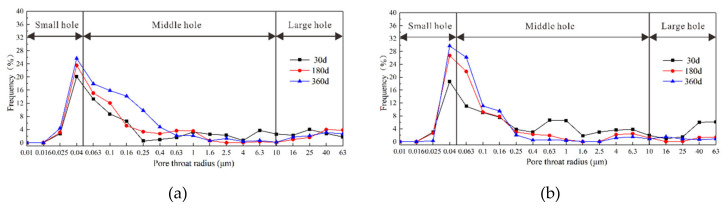

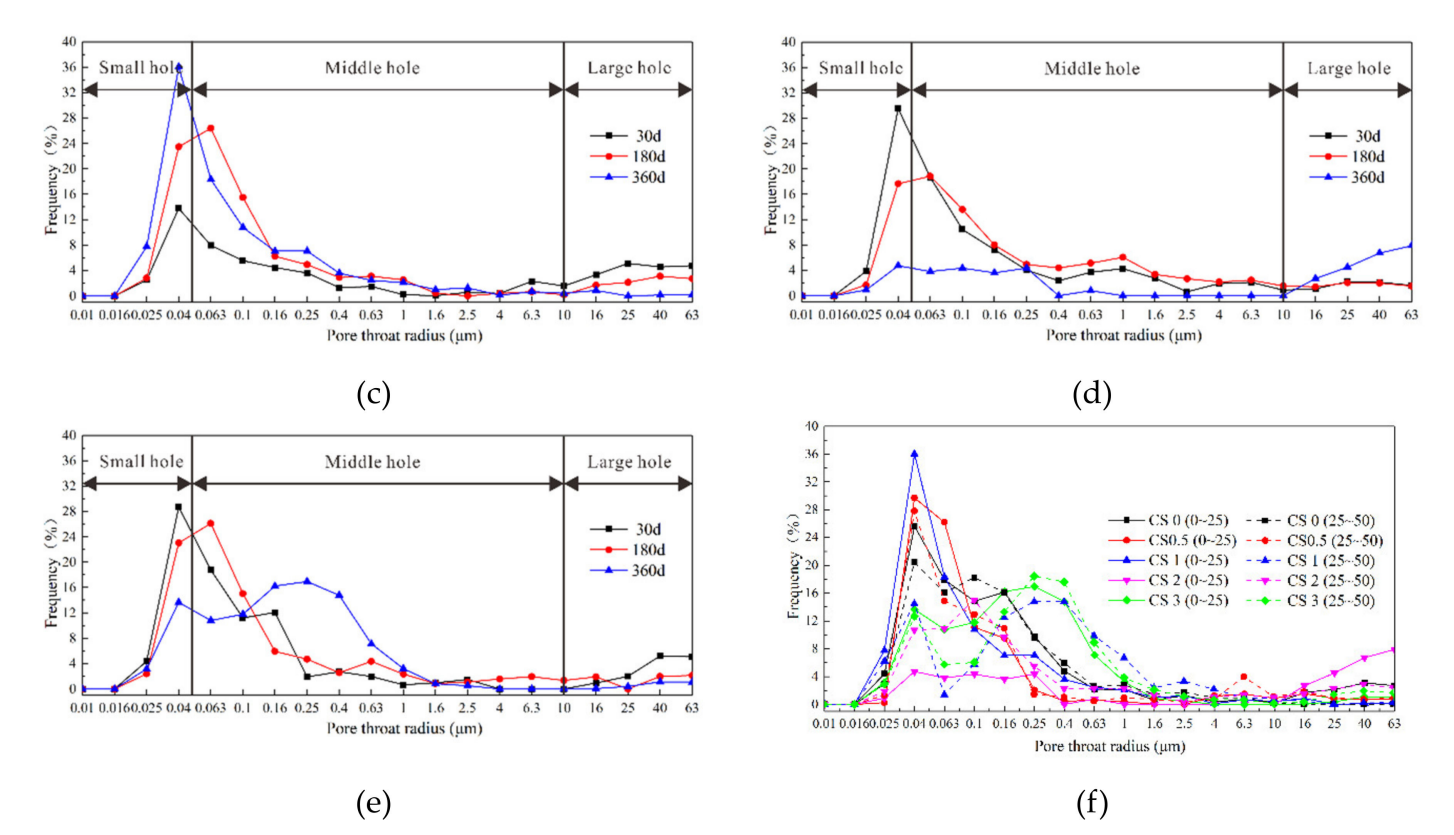
Evolution of concrete porosity after erosion by solution of different concentration: (**a**) CS0, (**b**) CS0.5, (**c**) CS1, (**d**) CS2, (**e**) CS3, (**f**) different depths after 360 d erosion.

The distribution of pores 25 mm from sample surfaces after NaCl-Na_2_SO_4_ solution corrosion for 30, 180, or 360 d showed that the proportions of small, middle, and large pores of in the CS0 samples did not change significantly after different exposure times (Figure 14). The relative proportions of pores followed the order: middle pores > small pores > large pores. With increased exposure time, the proportion of middle pores and small pores increased slightly. In the CS0.5 and CS1 samples, as the exposure time increased, the proportion of small pores and middle pores gradually increased, and the proportion of large pores decreased significantly. With higher Na_2_SO_4_ concentration (e.g., CS2), the proportion of small pores and middle pores gradually decreased over time. After 360 d, the proportion of large pores had increased significantly, and the original small and middle pores were “smoothed”. Upon further increased Na_2_SO_4_ (e.g., CS3 sample), the proportion of large pores decreased, and the proportion of small and medium pores increased up to 360 d. The pore distribution exhibited peaks in the range of small and medium pores, but the peaks corresponding to mesopores were more pronounced and spanned a larger distribution.

The distribution of pores at various depths after salt attack for 360 d showed that small and large pores in the CS0 samples were more common at depths between 0–25 mm than at depths of 25–50 mm, and mesopores showed the opposite trend (Figure 1b for pores at depths of 0–25 and 25–50 mm, and Figure 14f). In the CS0.5 and CS1 samples, the proportion of small and middle pores at depths of 0–25 mm was generally greater than that at depths of 25–50 mm, and there were more large pores at 025 mm depths than at 25–50 mm depths. There were fewer small and medium pores present in the 0–25 mm depth range of the CS2 samples than in the 25–50 mm depth range, and the proportion of large pores showed the opposite trend. Compared with the CS2 samples, the CS3 sample had fewer large pores and more middle and small pores. The proportion of large pores in the CS3 samples at depths of 0–25 mm was lower than the number of large pores at 25–50 mm depths, and the proportion of small and middle pores showed no appreciable change with sample depth.

These experimental results showed that the impact of NaCl in the salt solution on the concrete sample pore structure was not clear. With increased Na_2_SO_4_ concentration and corrosion time, the corrosion products (i.e., Aft and Gyp) gradually accumulated and filled the pores, cracks, and ITZ, thereby changing the pore distributions and allowing a transition from large pores to middle and small pores. As the Na_2_SO_4_ concentration, the crystal stress generated by the corrosion products caused small pores in a given sample to transform into large pores. Therefore, changes in the proportion of pore size further affected the diffusion of Cl^−^ and SO_4_^2^^−^ ions and the mechanical properties of the concrete.

## 4. Discussion

In this study, the effects of NaCl (at a constant concentration), Na_2_SO_4_ (at a range concentrations) and salt solution exposure times were analyzed by examining changes in the appearance, weight, compressive strength, RDEM, and Cl^−^ and SO_4_^2^^−^ diffusion depth in concrete samples. The depth of salt corrosion of concrete was observed, and a deterioration mechanism was proposed. XRD, SEM, EDS, and YG-97A capacitive mercury porosimeter analyses revealed changes in concrete minerals composition, microstructure, and pores contents after exposure to different salt solutions.

### 4.1. Mechanical Properties

During attack to different sodium salt solution, Cl^−^ and SO_4_^2^^−^ gradually migrated into the concrete samples through pores and cracks. Differences in concentration gradient, pores, and cracks were the main factor affecting ion diffusion. When the Na_2_SO_4_ concentration was ≤ 5%, as time passed or Na_2_SO_4_ increased, NaCl reacted with C-S-H and AFm to form Friedel’s salt and simultaneously release SO_4_^2^^−^. Meanwhile, Na_2_SO_4_ reacted with cement hydration products of AFm Friedel’s salt to form the corrosion product Aft. Silicate and C-S-H in the concrete are required to provide Ca^2+^. During this process, C-S-H gradually shifted from a block to a honeycomb structure, which had lower strength. The corrosion products (Aft and Friedel’s salt) filled the pores, cracks, and ITZ to certain. When the proportion of large holes in the sample decreased, the proportion of middle and small pores increased, and the concrete density increased. The crystal stress produced by the corrosion products was much smaller than the tensile strength of the matrix, and no new cracks generated. Coupled with the hardening effect of the cement, the mechanical properties (e.g., compressive strength and RDEM) of the concrete were improved as a result of the increased density.

When the salt solution contained more Na_2_SO_4_ (≥10 wt%), as the attack time increased, corrosion products accumulated, and the concrete density during the early stages was higher than that in the low salt concentration samples. As a result, the mechanical properties were improved, and overall concrete quality increased. When a higher salt solution continued to corrode the samples, numerous Aft, Gyp, and Glauber’s salt crystals appeared inside. The same results were observed via XRD and SEM analyses. At this time, the corrosion products in pores and ITZ generated larger crystal stresses than the tensile strength of the matrix, which widened and extended the original concrete cracks until a large number of connected pores appeared and intersecting cracks appeared [53,54]. In addition, the C-S-H blocks in the concrete gradually developed a network structure, which led to the gradual transition from small pores to large pores in the later stage of attack to more concentrated Na_2_SO_4_ solutions. As a certain depth from the concrete surface, the ITZ widened, and new cracks were generated, thus decreasing the concrete’s mechanical properties and weight.

### 4.2. Migration of Cl^−^

Following exposure to various salt solution concentrations for different periods of time, the Cl^−^ diffusion trends revealed that early in the exposure, the Cl^−^ content in the concrete only exposure to NaCl was the highest, and as the Na_2_SO_4_ concentration increased, the produced Friedel’s salt hindered Cl^−^ migration. However, Cl^−^ diffusion was promoted in the late stage of corrosion by high-concentration NaCl-Na_2_SO_4_ solution.

Analysis of Cl^−^ diffusion at different concrete sample depths after exposure to NaCl-Na_2_SO_4_ solution showed that in the early stage, the concentration gradients inside the solution and concrete were quite different; Cl^−^ and SO_4_^2^^−^ migrate rapidly, and the diffusion speed of Cl^−^ faster than that of SO_4_^2^^−^ [6,55]. As the Na_2_SO_4_ concentration in solution increased, the diffusion rate of SO_4_^2^^−^ also increased. As the exposure time increased, Cl^−^ ions in the CS0 samples reacted with aluminate (i.e., Afm) to form a small amount of Friedel’s salt, which filled the interior, thus slightly hindering the migration of Cl^−^.

At low Na_2_SO_4_ concentrations (≤ 5 wt%), Cl^−^ and SO_4_^2^^−^ gradually migrated into the concrete; however, SO_4_^2^^−^ preferentially reacted with C-S-H, CH, or Friedel’s salt to form Aft, and Friedel’s salt was effectively corroded away by Na_2_SO_4_. A portion of the Cl^−^ was also released to form free Cl^−^ [56,57,58], causing the Cl^−^ concentration in the pores to increase. At the same time, corrosion products filled the concrete pores, cracks, and ITZ, which changed the pore distribution in these samples, specifically, this caused the transition from large pores to middle and small pores, thereby hindering Cl^−^ migration. In the Cl^−^ diffusion process, corrosion products had a greater effect on Cl^−^ than the concentration gradient in the pores, ultimately, the Cl^−^ content in the CS0 samples was higher than that in the CS0.5 and CS1 samples.

Examination of the samples attacked to higher Na_2_SO_4_ concentrations (≥10 wt%) indicated that in the initial stage of exposure, with increased time and Na_2_SO_4_ concentration, the pores, cracks, and ITZ were completely filled with Aft, Gyp, and Na_2_SO_4_ crystals, thus slowing the Cl^−^ migration. However, due to the high Na_2_SO_4_ content in solution, the internal pores of the concrete released more Cl^−^ ions, thus increasing the concentration gradient between the internal pores, which help the diffusion of Cl^−^ ions in the concrete. As the attack continued, Na_2_SO_4_ corrosion products completely filled the pores and ITZ. At this point, crystal stresses generated by the corrosion products was less than the tensile force of the matrix, so no new microcracks were generated in the concrete. This was confirmed by the fact that the Cl^−^ ion content at a depth 2.5 mm of in the CS2 sample was the smallest when the erosion occurred for 270 d. As the Na_2_SO_4_ concentration increased further, the corrosion products generated in the CS3 samples continued to accumulate, thereby, increasing the crystal stresses beyond the tensile stress of the matrix. This resulted in the production of new pores and cracks inside the sample, and was confirmed based on the fact that small and middle pores were occupied. As the salt ratio decreased, the proportion of large pores increased. Highly concentrated Na_2_SO_4_ solution (≥15 wt%) created new Cl^−^ diffusion channels in the concrete samples attacked for 360 d. In addition, high Na_2_SO_4_ concentration released more Cl^−^ ions, thus cresting comparable Cl^−^ concentration in pores and cracks. Under the action of multiple factors (e.g., concentration gradient, cracks, and pores), the Cl^−^ in high concentrated Na_2_SO_4_ solution more easily diffused into the concrete samples. This was evidenced by the fact that the Cl^−^ content was the highest at a depth 2.5 mm depth after exposure to CS3 solution for 360 d. Through these analyses, it was determined that under high Na_2_SO_4_ concentrations (≥10 wt%), the initial stage of exposure hindered Cl^−^ diffusion. However, with increased concentration and exposure time, high Na_2_SO_4_ concentration facilitated Cl^−^ diffusion.

**Figure 15:**
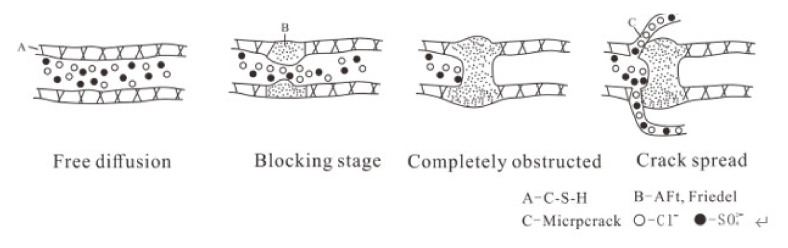
Cl^−^ diffusion process in pores.

Based on the above observations and analyses presented herein, after exposure to solution containing 5 wt% NaCl-Na_2_SO_4_ at 0, 2.5, 5, 10, or 15 wt%, Cl^−^ diffusion into the pores and reacted in concrete samples in four stages (Figure 15): free diffusion, hindering stage, complete hindering stage, and cracking stage.

## 5. Conclusions

This article discussed multiple factors contributing to the reduction in mechanical properties of concrete and the Cl^−^ content at various depths after exposure of solutions containing 5 wt% NaCl and 0, 2.5, 5, 10, and wt 15% Na_2_SO_4_ for different periods of time.

Exposure to low-concentration of NaCl (5 wt%)-Na_2_SO_4_ (≤ 5 wt%), as the Na_2_SO_4_ concentration and exposure time increased, the mass, compressive strength, and RDEM gradually increased, and large pores gradually transformed to middle and small pores.

In solution with higher concentration of NaCl (5 wt%)-Na_2_SO_4_ (≥10 wt%), the compressive strength, RDEM, and mass of concrete samples increased with increased Na_2_SO_4_ concentration, but there was a downward trend after attack for a certain period of time.

During the corrosion process by NaCl-Na_2_SO_4_ solutions, the migration speed of Cl^−^ was higher than that of SO_4_^2^^−^.

The Cl^−^ diffusion rate was related to the Na_2_SO_4_ concentration. Specifically, in an environment with a lower Na_2_SO_4_ concentration, Cl^−^, SO_4_^2^^−^ and cement hydration products hindered Cl^−^ migration. In a more concentration Na_2_SO_4_ environment, the Cl^−^ diffusion rate was hindered during the early stage of exposure, and the diffusion rate was promoted following longer corrosion periods.

## Figures and Tables

**Table 1:** Chemical composition of cementitious materials.

Chemical Composition	Al_2_O_3_	SiO_2_	SO_3_	Cl	TiO_2_	Fe_2_O_3_	Na_2_O	K_2_O	MgO	CaO
Content (%)	5.08	20.1	2.02	0.028	0.341	2.94	0.700	0.350	1.50	60.7

**Table 2:** Mixture proportion of concrete in the present study.

Concrete Grade	w/c	Cement (kg/m^3^)	Sand (kg/m^3^)	Gravel (kg/m^3^)	Water (kg/m^3^)
C35	0.4848	402.16	631	1199	195

**Table 3:** Corrosion conditions of specimens.

Specimen	Corrosive Environment		Specimen	Corrosive Environment	
	NaCl (wt%)	Na_2_SO_4_ (wt%)		NaCl (wt%)	Na_2_SO_4_ (wt%)
CS0	5%	0	CS2	5%	10%
CS0.5	5%	2.5%	CS3	5%	15%
CS1	5%	5%			

## Data Availability

Data sharing not applicable.
